# Quantitative anatomy of the growing quadratus lumborum in the human foetus

**DOI:** 10.1007/s00276-017-1901-4

**Published:** 2017-07-29

**Authors:** Magdalena Grzonkowska, Mariusz Baumgart, Mateusz Badura, Małgorzata Dombek, Marcin Wiśniewski, Monika Paruszewska-Achtel, Michał Szpinda

**Affiliations:** Department of Normal Anatomy, The Ludwik Rydygier Collegium Medicum in Bydgoszcz, The Nicolaus Copernicus University in Toruń, Łukasiewicza 1 Street, 85-821 Bydgoszcz, Poland

**Keywords:** Quadratus lumborum, Width, Length, Surface area, Cross-sectional area, Regression analysis, Human foetuses

## Abstract

**Purposes:**

The purpose of the study was to quantitatively evaluate the size of the quadratus lumborum and to precisely display its growth dynamics in the human foetus.

**Materials and methods:**

Using anatomical dissection, digital-image analysis (NIS Elements AR 3.0) and statistical analysis (Student’s *t* test, regression analysis), the length, width, surface area, and cross-sectional area of the quadratus lumborum were measured, and the width-to-length ratio was calculated in 58 human foetuses of both sexes (26♂, 32♀) aged 16–27 weeks.

**Results:**

Neither sex nor right-left significant differences were found in relation with the numerical data of the growing quadratus lumborum. The length, width, and cross-sectional area of the quadratus lumborum muscle increased logarithmically, while its surface area increased proportionately to fetal age. The following growth models were computed for the quadratus lumborum: *y* = −70.397 + 68.501 × ln(age) ± 1.170 for length, *y* = −20.435 + 8.815 × ln(age) ± 0.703 for width, *y* = −196.035 + 14.838 × age ± 13.745 for surface area, and *y* = −48.958 + 20.909 × ln(age) ± 1.100 for cross-sectional area.

**Conclusions:**

The fetal quadratus lumborum reveals neither sex nor bilateral differences. An increase in length and width of the growing quadratus lumborum follows in a commensurate fashion. The quadratus lumborum grows logarithmically with respect to its length, width, and cross-sectional area, and proportionately to age with respect to its surface area.

## Introduction

The quadratus lumborum muscle is located laterally in relation with the lumbar spine and spreads between rib XII and the iliac crest [17]. The muscle originates from the inner lip of the iliac crest and the iliolumbar ligament, and inserts into the transverse processes of the L_1_–L_4_ vertebrae and the inner surface of rib XII. Its dual innervation is derived from both short branches of the lumbar plexus and the subcostal, iliohypogastric, and ilioinguinal nerves [9, 26]. Incidentally, the quadratus lumborum is anteriorly crossed by the subcostal, iliohypogastric, and ilioinguinal nerves [26].

Due to the muscle’s position and its flattened and quadrilateral shape, its unilateral contraction allows lateral flexion of the spine, while its bilateral contraction supports the extension movement and also exerts a stabilizing effect on the lumbar spine. Furthermore, due to its insertion into rib XII, the quadratus lumborum exerts an auxiliary expiratory effect [17]. According to Hsu et al. [12], the quadratus lumborum, multifidus muscle, and erector spinae are all antagonists of the rectus abdominis muscle. The quadratus lumborum along with the gluteus medius allows maintaining the upright position and helps to stabilize the pelvis when transferring the weight from one leg to the other during gait. The quadratus lumborum also pulls the iliac ala towards the thorax which allows transferring a limb without touching the ground during gait [9].

Precise numerical data on the quantitative anatomy of the quadratus lumborum can be useful in anesthesiology for performing the so-called quadratus lumborum block. The local anesthetic injected through either the lateral or posterior approaches to the quadratus lumborum muscle may play a considerable role in perioperative pain management for abdominal surgery in children and adults, e.g., laparoscopy, colostomy, pyeloplasty, caesarean section, gastrostomy, and myocutaneous flap surgery [5, 16, 25].

The quadratus lumborum block results in the spread of the local anesthetic in the transversus abdominis plane. It is conducive as both supplementary intraoperative and postoperative analgesic methods, the latter may be used as the main component of multimodal analgesia following abdominal surgery [25]. To date, however, linear, planar and spatial dimensions of the quadratus lumborum have been analyzed in adult individuals only [14, 18]. Therefore, this paper is the first report in the literature to perform a morphometric analysis of the quadratus lumborum in the human foetus.

In the present study, we aimed:

- to perform morphometric analysis of the quadratus lumborum (linear and planar parameters) in order to determine their reference age-specific values;
- to examine possible male–female and right–left differences for the analyzed parameters;
- to compute growth dynamics for the analyzed parameters, expressed by mathematical models best-matched to fetal age.

## Materials and methods

The study material comprised 58 human foetuses (26 males and 32 females) aged 16–27 weeks of gestation, originating from spontaneous abortions or preterm deliveries during the years 1989–2001 because of placental insufficiency. On macroscopic examination, both internal and external conspicuous anatomical malformations, including those related to chromosomal disorders, were ruled out in all included specimens, thus being identified as normal. The fetuses did not display any developmental abnormalities, particularly including the musculoskeletal system. The experiment was approved by the Bioethics Committee of our university (KB 186/2016). The fetal age was determined on the crown-rump length [13] and the known date of the beginning of the last maternal menstrual period. Furthermore, the fetuses studied could not suffer from growth retardation, as the correlation between the gestational age based on the crown-rump length (CRL) and that calculated by the last menstruation attained the value *R* = 0.98 (*p* < 0.001). Table 1 lists the characteristics of the study group, including age, number, and sex of the foetuses.

**Table 1:** Age, number, and sex of the fetuses studied

Gestational age	Crown-rump length (mm)				Number of fetuses	Sex	
Weeks (Hbd-life)	Mean	SD	Min.	Max.	*N*	♂	♀
16	107.0	8.49	101	113	2	2	0
17	122.0	3.61	118	125	3	2	1
18	134.1	4.56	130	142	10	5	5
19	147.7	4.13	143	152	6	2	4
20	162.1	4.85	156	168	7	1	6
21	173.3	2.96	169	179	9	5	4
22	183.2	2.77	181	188	5	2	3
23	201.0	0.00	201	201	2	1	1
24	208.5	2.95	205	211	6	2	4
25	217.7	2.52	215	220	3	2	1
26	230.5	2.12	229	232	2	1	1
27	236.7	2.89	235	240	3	1	2
Total					58	26	32

**Fig. 1:**
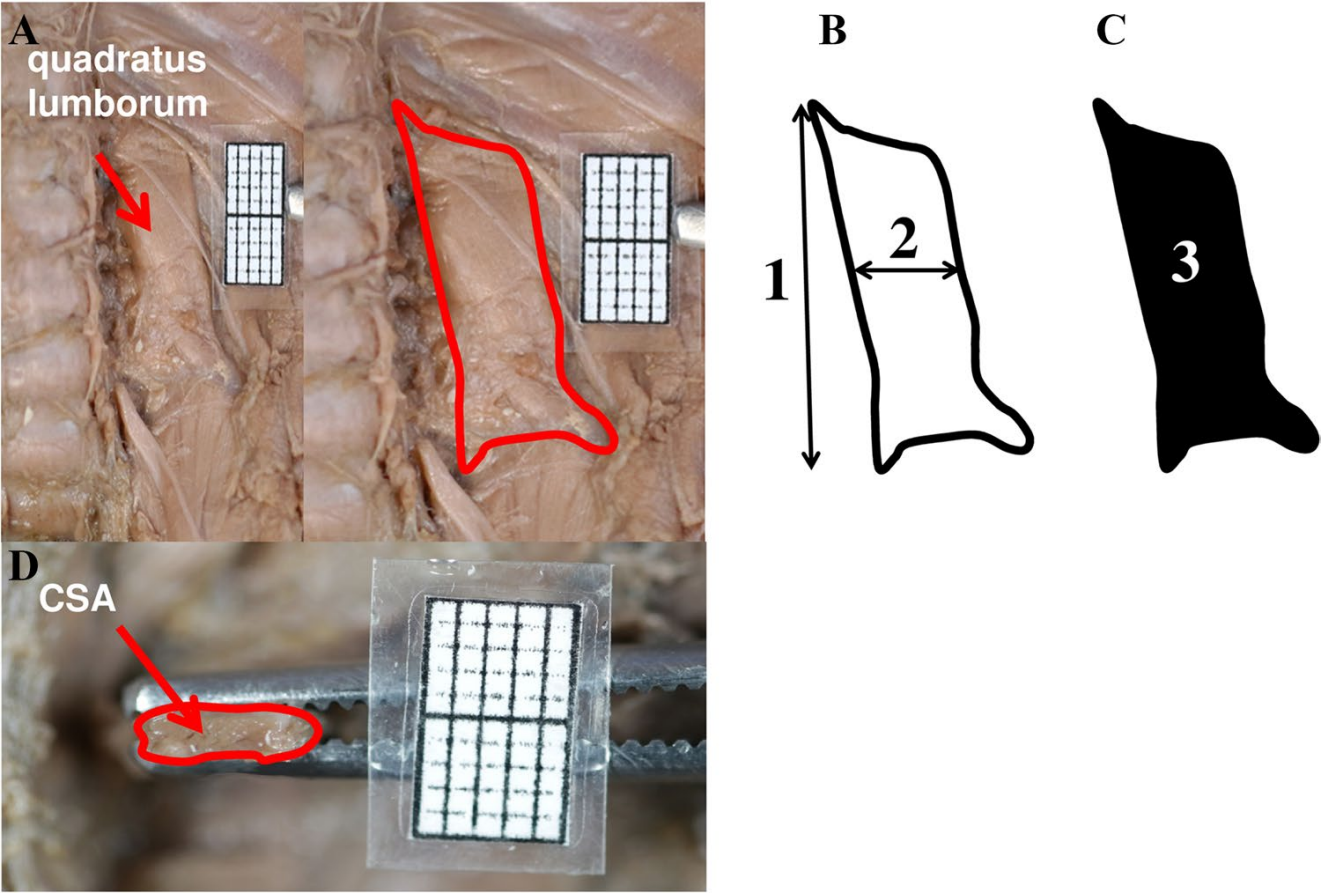
Contoured quadratus lumborum muscle (**a**) in a female foetus at 22 weeks showing the measured parameters (**b**, **c**): *1* length, *2* width, and *3* surface area, and cross-sectional area **(d)**

All foetuses were preserved by having been immersed in 10% neutral formalin solution. Functionally, such formalin-fixed skeletal muscles exist in a state of partial contraction, just in the middle between fully relaxed and contracted skeletal muscles of a living individual [15]. Of note, a significant decrease in muscle length occurs when isolated muscles are subjected to fixation. This remains in stark contrast to our study, in which skeletal musculature was fixed “in situ”, i.e., on the skeleton [7]. In other words, the foetuses had been preserved in formalin solution before the “in situ” muscles were examined, thus reducing muscle shrinkage by only 0.5–1.0% [3, 7]. Each quadratus lumborum muscle was anatomically dissected so as to completely visualize it from its origin to its insertion, and recorded with a millimeter scale, using a camera of Canon EOS 70D(W). The digital pictures of the quadratus lumborum were quantitatively evaluated with the use of digital-image analysis (NIS Elements AR 3.0), which semi-automatically estimated all the parameters studied. The digital method enabled to precisely estimate all measurements with an accuracy of 0.1 mm. Finally, each muscle at its midway was horizontally cut so as to measure its cross-sectional area. In every quadratus lumborum, the following five parameters were measured (Fig. 1):

1. Length (mm)—based on the distance between its origin and insertion;
2. Width (mm)—based on the distance between its medial and lateral borderlines, measured at its midway;
3. Surface area (mm^2^)—based on its area outlined;
4. Cross-sectional area (mm^2^)—based on its area outlined, after having been cut at its midway; and
5. Width-to-length ratio—the quotient of the muscle width and length.

All measurements were carried out by two independent researchers (M.G., M.B.). Each measurement was performed three times under the same conditions but at different times, and then averaged. The differences between repeated measurements, as the intra-observer variation, were assessed by the one-way ANOVA test for paired data and post hoc RIR Tukey test. Thus, to examine inter-observer reproducibility, intra-class correlation coefficients (ICC) were calculated. The best ICC turned out to be ICC (2,1). In the present study to analyze all the numerical data we used the Statistica 12.5 and PQStat 1.6.2. programs. The results were expressed as arithmetic means with standard deviations (SD). To compare the means, Student’s *t* test for dependent (left–right) and independent (male–female) variables, and one-way analysis of variance were used. The characterization of developmental dynamics of the analyzed parameters was based on linear and curvilinear regression analysis. The match between the estimated curves and measurement results was grounded in the coefficient of determination (*R* ^2^). Differences were considered statistically significant at *p* < 0.05.

## Results

**Fig. 2:**
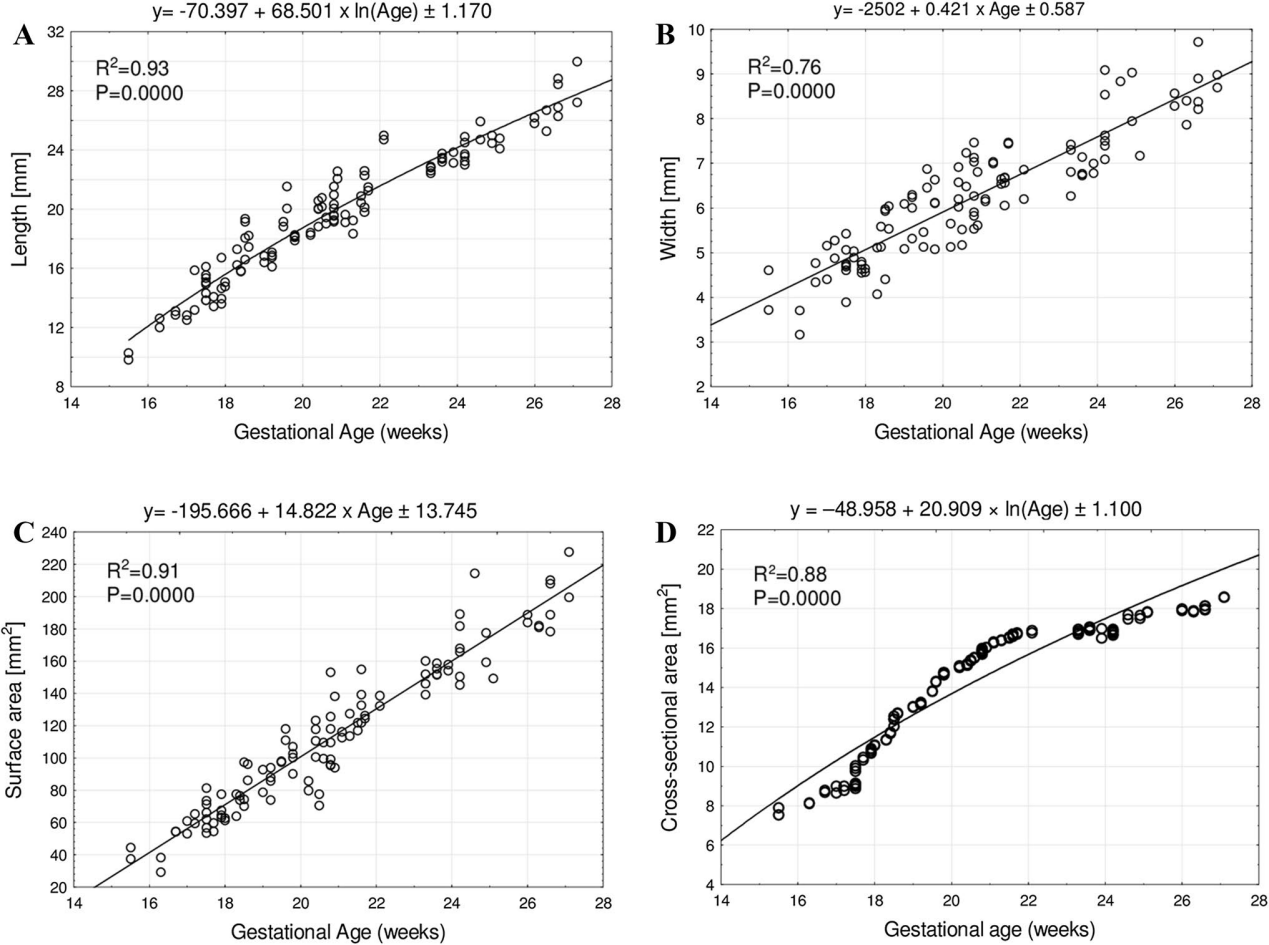
Regression lines for length (**a**), width (**b**), surface area (**c**), and cross-sectional area (**d**) of the quadratus lumborum muscle

In the foetuses studied, we did not find any explicit anomalies of the quadratus lumborum muscle such as partial or complete agenesis, incompletely developed muscle, shortening of the muscle, or its atrophy. As displayed in Table 2, the intra-class correlation coefficients (ICC) calculated on the base of two independent observers were statistically significant (*p* < 0.001) and of excellent reproducibility. As exposed in Table 3, the statistical analysis revealed neither sex nor bilateral differences for all analyzed parameters (*p* > 0.05). Therefore, we investigated the developmental dynamics of the four established parameters without taking sex or side into account. Tables 4 and 5 summarize the means and SD for length, width, surface area, and cross-sectional area of the growing quadratus lumborum. Table 6 presents the mean values of the width-to-length ratio, on average of 0.32 ± 0.04. This supported a commensurate increase in length and width of the growing quadratus lumborum throughout the study period. The growth dynamics of the length, width, and cross-sectional area of the quadratus lumborum muscle followed the logarithmic functions *y* = −70.397 + 68.501 × ln(age) ± 1.170, *y* = −20.435 + 8.815 × ln(age) ± 0.703, and *y* = −48.958 + 20.909 × ln(age) ± 1.100, respectively. The surface area of the quadratus lumborum followed the linear function *y* = −196.035 + 14.838 × age ± 13.745 (Fig. 2).

**Table 2:** Intra-class correlation coefficient (ICC) values for inter-observer reproducibility

Parameter	ICC (2,1)
Length	0.999*
Width	0.988*
Surface area	0.997*
Cross-sectional area	0.991*

Intra-class correlation coefficients marked with * are statistically significant at *p* < 0.0001

**Table 3:** Statistical analysis of numerical data (mean ± SD) of the quadratus lumborum in relation with the side and sex

Muscle parameter	Side	♂	SD	♀	SD	*p*
Length (mm)	Right	19.24	4.66	20.06	4.27	0.639
	Left	19.06	4.66	19.97	3.96	0.390
Width (mm)	Right	6.15	1.58	6.21	1.27	0.247
	Left	6.30	1.68	6.29	1.25	0.119
Surface area (mm^2^)	Right	109.20	48.98	114.23	43.17	0.500
	Left	110.23	51.11	116.39	43.65	0.401
Cross-sectional area (mm^2^)	Right	13.79	3.65	14.71	2.68	0.104
	Left	13.83	3.62	14.72	2.67	0.107

**Table 4:** Length and width of the quadratus lumborum muscle

Gestational age (weeks)	Number of fetuses	Length (mm)					Width (mm)				
		Right		Left		*p*	Right		Left		*p*
		Mean	SD	Mean	SD		Mean	SD	Mean	SD	
16	2	11.13	1.23	11.23	1.97	>0.05	3.45	0.39	4.15	0.64	>0.05
17	3	12.86	0.35	13.93	1.69	>0.05	4.79	0.41	4.81	0.44	>0.05
18	10	15.24	1.26	14.81	0.88	>0.05	4.71	0.48	4.73	0.53	>0.05
19	6	17.28	1.16	17.53	1.05	>0.05	5.80	0.43	5.88	0.94	>0.05
20	7	19.36	1.27	18.96	1.00	>0.05	6.07	0.72	5.98	0.61	>0.05
21	9	20.18	1.20	19.91	1.12	>0.05	6.12	0.63	6.41	1.01	>0.05
22	5	21.75	2.03	21.96	1.78	>0.05	6.69	0.51	6.74	0.46	>0.05
23	2	22.74	0.22	22.62	0.25	>0.05	6.79	0.73	7.11	0.44	>0.05
24	6	23.55	0.49	23.75	0.67	>0.05	7.39	0.67	7.34	0.89	>0.05
25	3	25.06	0.78	24.60	0.44	>0.05	7.81	1.95	8.32	1.37	>0.05
26	2	26.45	0.34	25.55	0.36	>0.05	8.35	0.09	8.22	0.49	>0.05
27	3	28.24	1.86	27.66	1.04	>0.05	8.75	0.33	8.88	0.77	>0.05

**Table 5:** Surface area and cross-sectional area of the quadratus lumborum muscle

Gestational age (weeks)	Number of fetuses	Surface area (mm^2^)					Cross-sectional area (mm^2^)				
		Right		Left		*p*	Right		Left		*p*
		Mean	SD	Mean	SD		Mean	SD	Mean	SD	
16	2	33.47	5.86	41.37	44.43	>0.05	7.83	0.42	8.03	0.18	>0.05
17	3	58.34	3.36	57.63	66.84	>0.05	8.92	0.12	8.70	0.07	>0.05
18	10	67.27	9.36	64.58	88.57	>0.05	10.31	1.00	10.39	0.92	>0.05
19	6	85.06	8.02	87.40	12.04	>0.05	12.73	0.46	12.85	0.34	>0.05
20	7	104.46	13.41	101.70	12.62	>0.05	14.69	0.49	14.71	0.50	>0.05
21	9	105.91	18.48	111.09	22.68	>0.05	15.89	0.32	15.88	0.34	>0.05
22	5	127.84	7.50	134.00	14.26	>0.05	16.67	0.12	16.70	0.12	>0.05
23	2	148.97	4.13	149.71	14.47	>0.05	16.87	0.13	16.79	0.12	>0.05
24	6	160.21	12.38	161.43	15.14	>0.05	16.87	0.13	16.83	0.21	>0.05
25	3	175.15	34.03	175.96	25.65	>0.05	17.66	0.15	17.66	0.19	>0.05
26	2	183.14	1.33	184.69	55.53	>0.05	17.95	0.06	17.86	0.04	>0.05
27	3	198.31	26.14	205.92	55.60	>0.05	18.21	0.32	18.24	0.31	>0.05

**Table 6:** Quadratus lumborum muscle length-to-width ratio

Gestational age (weeks)	Length-to-width ratio	SD	Min	Max
16	0.35	0.09	0.26	0.47
17	0.36	0.03	0.33	0.41
18	0.32	0.04	0.23	0.36
19	0.34	0.04	0.24	0.39
20	0.31	0.03	0.27	0.37
21	0.31	0.05	0.21	0.38
22	0.31	0.03	0.25	0.35
23	0.31	0.02	0.27	0.33
24	0.31	0.03	0.28	0.36
25	0.32	0.06	0.22	0.40
26	0.32	0.01	0.31	0.33
27	0.32	0.03	0.28	0.36

## Discussion

Increased unilateral or bilateral tonus of the quadratus lumborum frequently occurs in patients with lower back pain. Chronic contraction of the muscle within the lower back contributes to the transfer of the tonus via the thoracodorsal fascia to the shoulder girdle and neck. Postural defects such as hyperkyphosis may cause an increase in the tonus of the paravertebral muscles, such as the quadratus lumborum, multifidus, erector spinae, or levator scapulae muscles. This is due to the tendency of the body to restore and maintain the correct center of gravity [9].

The quadratus lumborum is of relevance in anesthesiology. The quadratus lumborum block was first described as late as in 2007 by Dr. Rafael Blanco. A local anesthetic can be administered into the anterolateral (type I) or posterior (type II) edges of the quadratus lumborum, resulting in a wide distribution of the drug into the paravertebral space, providing a long-term analgesia after surgical treatment in the abdominal area [4, 18]. The quadratus lumborum block allows anesthesia of the area covered by dermatomes T_4_ to L_2_ [6]. The anesthetic passes into the paravertebral space or is distributed along the surrounding blood vessels, lymphatics, and nerves [26].

In this study, we demonstrated that in terms of morphometric parameters, the quadratus lumborum did not demonstrate any sex or right–left differences. Of note, neither sex nor right–left differences were found by the authors dealing with quantitative anatomy of other skeletal muscles in the human foetus, i.e., triceps brachii [10], biceps brachii [23], trapezius [1], deltoid [22], biceps femoris [24], semimembranosus [2], and semitendinosus [3] muscles. It should be emphasized that both the length and width of the growing quadratus lumborum grew proportionately, since the width-to-length ratio was relatively constant, reaching the value of 0.32 ± 0.04. The length of the quadratus lumborum muscle in adults was evaluated on autopsy material by Delp et al. [8]. These authors measured the total (musculotendinous) length and the muscle length of the anterior and posterior layers that were 11.7 ± 1.7, 9.3 ± 1.3, 10.7 ± 1.3, and 8.1 ± 1.2 cm, respectively. However, the aforementioned study was conducted in a small group, consisting of five subjects only. Regrettably, basing on these literature data, we could not calculate the width-to-length ratio, because the article in question had solely been focused on the length of the quadratus lumborum muscle in five adult individuals, without taking into account the muscle width. In their study, the authors observed that the anterior section of the quadratus lumborum consisted of both iliocostal and iliothoracic fibers, the middle section consisted of lumbocostal fibers, while the posterior section comprised both iliolumbar and iliocostal fibers. Moreover, the authors found the quadratus lumborum to primarily function as an expiratory muscle, since more than half of its fibers referred to costal ones. The quadratus lumborum muscle is also to stabilize rib XII and to affect the static parameters of the diaphragm. Stark et al. [21] measured the length of the muscular and tendinous sections of the quadratus lumborum; however, these authors obtained lower values than those achieved by Delp et al. [8]. The muscle and tendon lengths were 6.0 ± 2.1 and 5.5 ± 2.3 cm on the right and 3.2 ± 1.4 and 3.3 ± 1.6 cm on the left, respectively.

Since the quadratus lumborum is a typical quadrangular muscle, its anatomical cross-sectional area obtained in the present study is equal to the so-called physiological cross-sectional area. Of note, the physiological cross-sectional area of any skeletal muscle prerequisites its forces generated. It is widely accepted that the maximum force that can be produced by a muscle is directly proportionate to its physiological cross-sectional area. Since the cross-sectional area of the quadratus lumborum muscle calculated in this study followed logarithmically, the force potentially exerted by the growing quadratus lumborum muscle must indubitably increase according to a natural logarithmic function. Regrettably, the professional literature is devoid of any numerical data concerning the cross-sectional area of quadratus lumborum in the foetus, thus limiting comprehensive discussion in this subject. However, some authors measured the cross-sectional area in adults with the use of MRI and CT. This was exemplified by Ranson et al. [18], who measured the cross-sectional area of the quadratus lumborum in professional cricket fast bowlers. These measurements were done at the four levels, corresponding to the lower edges of vertebrae L_1_–L_4_. The cross-sectional area of the right and left muscles was, respectively: 3.78 and 3.61 cm^2^ at level L_1_, 5.9 and 5.43 cm^2^ at level L_2_, 7.06 and 6.94 cm^2^ at level L_3_, and 9.22 and 8.25 cm^2^ at level L_4_. Furthermore, Hides et al. [11] assessed the cross-sectional area of the quadratus lumborum at the level of intervertebral disc L_3_–L_4_ for the dominant and non-dominant legs in professional football players. The study was conducted at three intervals of several months. The mean cross-sectional areas obtained in the first, second, and third measurements for the dominant and non-dominant legs were: 8.79 ± 0.29 and 9.90 ± 0.31 cm^2^, 8.84 ± 0.41 and 9.54 cm^2^, and 8.17 ± 0.22 and 9.31 ± 0.26 cm^2^, respectively. Surprisingly enough, the three cross-sectional areas of the quadratus lumborum were significantly smaller on the side of the dominant leg. The cross-sectional area of the quadratus lumborum was also measured using MRI by Hsu et al. [12] in healthy young individuals. At the L_4_ and L_5_ vertebrae, the muscle cross-sectional areas were 6.31 ± 3.01 and 3.74 ± 3.51 cm^2^, respectively. Kamaz et al. [14] measured the cross-sectional area of the quadratus lumborum with the use of CT in healthy women and women with chronic lumbar spine pain. The measurements were done both above and below vertebral body L_4_. The cross-sectional areas of the quadratus lumborum measured above vertebral body L_4_ in suffering and healthy women were 3.15 ± 0.94 and 3.63 ± 1.05 cm^2^, respectively, with a statistically significant difference. Of note, the values obtained below the L_4_ vertebral body were 3.77 ± 1.25 and 3.98 ± 1.17 cm^2^, respectively, with no statistical difference. Furthermore, Stark et al. [20] in their CT study with a 3D reconstruction of the quadratus lumborum found the mean cross-sectional area of the muscle on the left and right sides to be 2.4 ± 0.9 and 3.3 ± 1.5 cm^2^, respectively.

As far as the muscle volume is concerned, Sanchis-Moysi et al. [19] in an MRI study measured the volume of the quadratus lumborum in tennis players, football players, and people not practicing sports. The volumes referred to three equal segments (superior, middle, and inferior), spanning the distance from the L_1_–L_2_ intervertebral disc to the pubic symphysis. The volumes of the superior segment measured on the dominant and non-dominant sides were, respectively, 13.6 ± 5.8 and 13.0 ± 5.5 cm^3^ in tennis players, 16.2 ± 3.3 and 16.2 ± 3.3 cm^3^ in football players, and 12.1 ± 3.8 and 13.3 ± 3.3 cm^3^ in people not practicing sports. The volumes of the middle segment were, respectively, 22.4 ± 4.2 and 23.5 ± 5.0 cm^3^ in tennis players, 29.6 ± 5.5 and 29.6 ± 8.8 cm^3^ in football players, and 17.0 ± 4.5 and 20.5 ± 9.8 cm^3^ in people not practicing sports. The volumes of the inferior segments were, respectively, as follows: 33.5 ± 7.7 and 35.8 ± 9.3 cm^3^ in tennis players, 41.2 ± 9.6 and 39.2 ± 10.1 cm^3^ in football players, and 23.5 ± 5.3 and 27.2 ± 6.6 cm^3^ in people not practicing sports. Of note, the total volumes on the dominant and non-dominant sides were, respectively: 69.4 ± 14.6 and 72.2 ± 13.2 cm^3^ in tennis players, 86.9 ± 14.5 and 85.5 ± 19.6 cm^3^ in football players, and 52.3 ± 12.1 and 60.9 ± 19.1 cm^3^ in people not practicing sports. As it turned out, hypertrophy of the quadratus lumborum was demonstrated in the subjects practicing sports. According to Stark et al. [21], the mean muscle volumes on the right and left sides were 31.3 and 48.3 cm^3^, respectively.

It should be emphasized that this has been the first report in the professional literature to compute mathematical models as a function of fetal age for the growing quadratus lumborum. The natural growths were expressed by the following functions: *y* = −70.397 + 68.501 × ln(age) ± 1.170 for length, *y* = −20.435 + 8.815 × ln(age) ± 0.703 for width, and *y* = −48.958 + 20.909 × ln(age) ± 1.100 for cross-sectional area. The surface area of the growing quadratus lumborum increased proportionately to age, in accordance with the function: *y* = −196.035 + 14.838 × age ± 13.745. Age-specific reference data for normative values of the quadratus lumborum muscle in the human foetus may be conducive in detailed evaluation of both the skeletal system and fetal development, with potential relevance for surgical treatment and anesthesia in neonates. Regrettably, our numerical findings do not seem to be applied to the quadratus lumborum muscle in the adult.

## Conclusions

1. The fetal quadratus lumborum reveals neither sex nor bilateral differences.
2. An increase in length and width of the growing quadratus lumborum follows in a commensurate fashion.
3. The quadratus lumborum grows logarithmically with respect to its length, width and cross-sectional area, and proportionately to age with respect to its surface area.
